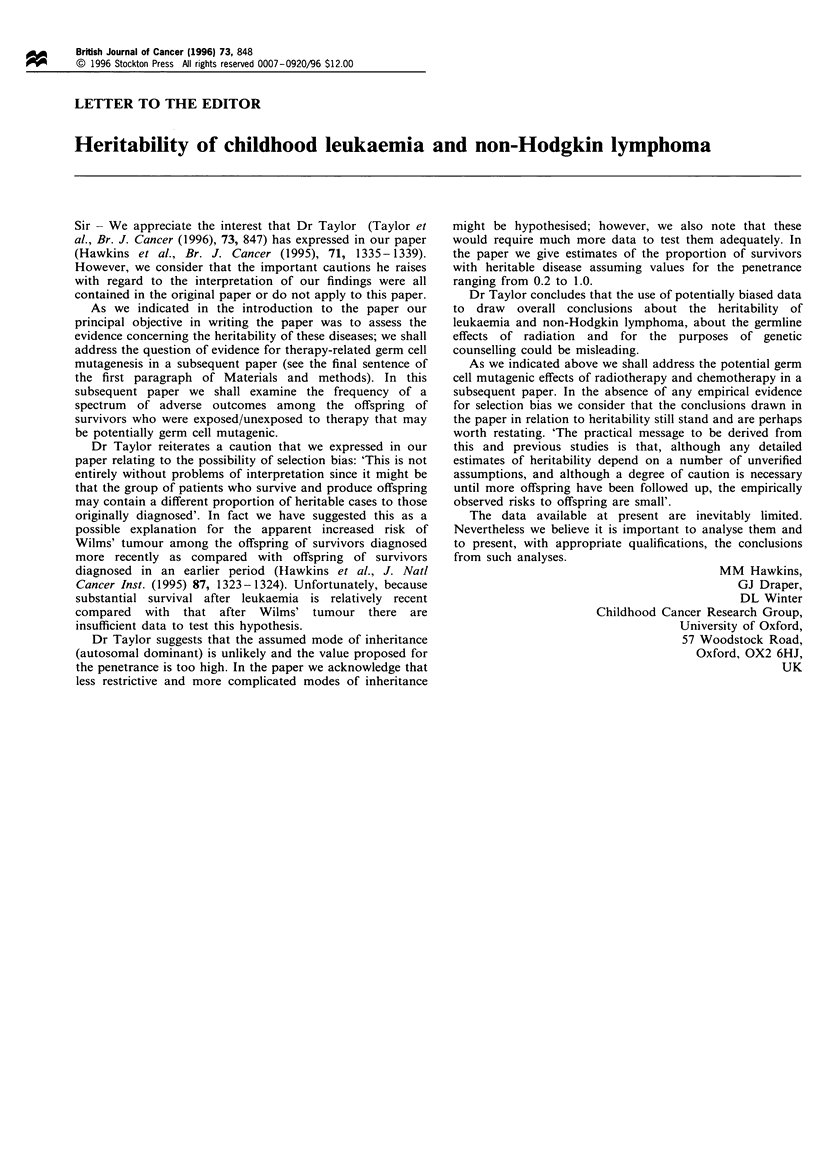# Heritability of childhood leukaemia and non-Hodgkin lymphoma

**Published:** 1996-03

**Authors:** M M Hawkins, G J Draper, D L Winter


British Journal of Cancer (1996) 73, 848
© 1996 Stockton Press All rights reserved 0007-0920/96 $12.00

LETTER TO THE EDITOR

### Heritability of childhood leukaemia and non-Hodgkin lymphoma

Sir – We appreciate the interest that Dr Taylor (Taylor *et al.*, *Br. J. Cancer* (1996), **73,** 847) has expressed in our paper (Hawkins *et al.*, *Br. J. Cancer* (1995), **71,** 1335–1339). However, we consider that the important cautions he raises with regard to the interpretation of our findings were all contained in the original paper or do not apply to this paper.

As we indicated in the introduction to the paper our principal objective in writing the paper was to assess the evidence concerning the heritability of these diseases; we shall address the question of evidence for therapy-related germ cell mutagenesis in a subsequent paper (see the final sentence of the first paragraph of Materials and methods). In this subsequent paper we shall examine the frequency of a spectrum of adverse outcomes among the offspring of survivors who were exposed/unexposed to therapy that may be potentially germ cell mutagenic.

Dr Taylor reiterates a caution that we expressed in our paper relating to the possibility of selection bias: 'This is not entirely without problems of interpretation since it might be that the group of patients who survive and produce offspring may contain a different proportion of heritable cases to those originally diagnosed'. In fact we have suggested this as a possible explanation for the apparent increased risk of Wilms' tumour among the offspring of survivors diagnosed more recently as compared with offspring of survivors diagnosed in an earlier period (Hawkins *et al.*, *J. Natl Cancer Inst.* (1995) **87,** 1323–1324). Unfortunately, because substantial survival after leukaemia is relatively recent compared with that after Wilms' tumour there are insufficient data to test this hypothesis.

Dr Taylor suggests that the assumed mode of inheritance (autosomal dominant) is unlikely and the value proposed for the penetrance is too high. In the paper we acknowledge that less restrictive and more complicated modes of inheritance might be hypothesised; however, we also note that these would require much more data to test them adequately. In the paper we give estimates of the proportion of survivors with heritable disease assuming values for the penetrance ranging from 0.2 to 1.0.

Dr Taylor concludes that the use of potentially biased data to draw overall conclusions about the heritability of leukaemia and non-Hodgkin lymphoma, about the germline effects of radiation and for the purposes of genetic counselling could be misleading.

As we indicated above we shall address the potential germ cell mutagenic effects of radiotherapy and chemotherapy in a subsequent paper. In the absence of any empirical evidence for selection bias we consider that the conclusions drawn in the paper in relation to heritability still stand and are perhaps worth restating. 'The practical message to be derived from this and previous studies is that, although any detailed estimates of heritability depend on a number of unverified assumptions, and although a degree of caution is necessary until more offspring have been followed up, the empirically observed risks to offspring are small'.

The data available at present are inevitably limited. Nevertheless we believe it is important to analyse them and to present, with appropriate qualifications, the conclusions from such analyses.

MM Hawkins,
GJ Draper,
DL Winter
Childhood Cancer Research Group,
University of Oxford,
57 Woodstock Road,
Oxford, OX2 6HJ,
UK